# Effects of Acute Altitude, Speed and Surface on Biomechanical Loading in Distance Running

**DOI:** 10.3390/s26010276

**Published:** 2026-01-01

**Authors:** Olaf Ueberschär, Marlene Riedl, Daniel Fleckenstein, Roberto Falz

**Affiliations:** 1 Department of Engineering and Industrial Design, Magdeburg-Stendal University of Applied Sciences, 39114 Magdeburg, Germany; marlene.riedl@partner.h2.de (M.R.); roberto.falz@h2.de (R.F.); 2 Institute for Applied Training Science, 04109 Leipzig, Germany; fleckenstein@iat.uni-leipzig.de; 3 Department of Orthopaedic and Trauma Surgery, Martin Luther University Halle-Wittenberg, 06120 Halle, Germany

**Keywords:** hypoxia, endurance running, triathlon, peak tibial acceleration, cadence, shock attenuation, running gait symmetry

## Abstract

Altitude training camps are a popular measure to enhance endurance performance at sea level. This study elucidates the effects of acute altitude-induced hypoxia, running speed and surface on cadence, peak tibial acceleration (PTA), gait asymmetry and residual shock in distance running. Ten healthy, trained native lowlanders (6 males, 4 females; 28.2 ± 9.2 years; mean ${\dot{V}}_{O_{2,peak}}$ of 54.9 ± 5.9 mL min^−1^ kg^−1^) participated in this study. They ran 1500 m bouts of at 50, 1000 and 2300 m above mean sea level on paved roads and natural trails at three different speeds. Those speeds were chosen to represent the most common training zones and were defined as $v_{1}=90\%\cdot v_{\mathrm{VT}1}$, $v_{2}=\frac{1}{2}\left(v_{\mathrm{VT}1}+v_{\mathrm{VT}2}\right)$ and $v_{3}=100\%\cdot v_{\mathrm{VT}2}$, with $v_{\mathrm{VT}1}$ and $v_{\mathrm{VT}2}$ denoting the speeds at the ventilatory thresholds 1 and 2. Based on the experimental results, cadence increased by +2.2 spm per +1 km h^−1^ (*p* < 0.001) and fell by −1.1. spm per +1000 m of elevation (*p* < 0.001), whereas surface did not show any significant effect. Likewise, PTA was not affected by surface, but grew by 0.9 *g* per +1 km h^−1^ (*p* < 0.001), and decreased by −0.6 *g* per +1000 m in elevation, with significant effects particularly at speeds beyond *v*_VT1_ (*p* < 0.049). Absolute lateral asymmetry was not altered by elevation, surface or running speed. Mean shock attenuation increased with running speed by +2.5 percentage points per +1 km h^−1^ (*p* < 0.001) but was independent of elevation and surface. In essence, running speed seems to be the predominant factor defining biomechanical loading, even under acute hypoxia and for varying surface conditions.

## 1. Introduction

Altitude training camps are a common and well-established measure in the training regimens of elite endurance athletes to utilise hypoxia-induced adaptations to enhance sea-level endurance performance [1,2,3,4,5,6,7,8], particularly of runners and triathletes [9,10]. These camps are typically held at moderate elevations between 2000 and 2500 metres above mean sea level (AMSL) [2,11]. Basically, exposure to reduced oxygen availability for a sufficiently long period of at least three weeks triggers increased production of erythropoietin and a consequent increase in total haemoglobin mass and the blood’s oxygen-carrying capacity, as well as a heightened capillary density, mitochondrial efficiency and buffering capacity in muscles [2,5,6,11,12]. At the same time, training intensities can be kept at an acceptable level. After returning to sea level, responsive athletes are likely to benefit from a temporary “performance window” of approximately 14 to 30 days [4,11,13].

Despite these potentially beneficial performance outcomes, training under altitude conditions is subject to the repercussions of altered physiological responses and biomechanical loads. For instance, a direct translation of intensity control from sea level to altitude conditions based on heart rate, lactate levels or external performance metrics (e.g., running speed or pedalling power output) may be challenging: On the one hand, aerobic capacity (i.e., peak oxygen uptake) and peak exercise heart rate have been shown to decrease under acute hypoxia [7,14,15], with effect sizes varying between specific exercises, e.g., running or cycling [14]. On the other hand, anaerobic capacity, i.e., lactate accumulation and tolerance at submaximal efforts, exhibits a duration-dependent different response [8,15], sometimes referred to as the “lactate paradox” [8]. Irrespective of the underlying details, it is evident that, owing to the generally altered physiological responses, factors defining exercise intensities (e.g., running speed, incline, etc. [16,17]) need to be carefully adjusted under altitude conditions to achieve a comparable training stimulus and avoid overreaching.

Given that the majority of popular altitude training camp sites are situated in rather rural mountain regions [10], running sessions often include off-road courses on hilly trails with natural surfaces. These courses may alter the biomechanical loading of the musculoskeletal system due to uphill and downhill segments, changes in speed and varying running surfaces [18,19,20,21,22,23]. Higher running speeds generally increase cadence (CAD, i.e., step frequency) and peak tibial acceleration (PTA) [16,20,23], while, conversely, they may potentially result in lower PTA asymmetry [18,24]. Regarding the effects of running surface on CAD and on PTA as an external biomechanical loading metric [25,26,27,28,29,30,31,32,33,34], the extant literature provides an inconclusive picture, with partly conflicting results that depend on surface properties [18,19,21,22,35,36]. A recent case study has demonstrated that topographical and surface-related running course conditions may alter PTA and lateral, i.e., between-legs asymmetry in PTA [19]. Potential effects on lateral asymmetry in running gait are of particular interest to the successful balancing of exercise loading and recovery, since asymmetry is regularly discussed as a possible risk factor for sustaining overuse injuries [37,38,39]. In light of this, special attention should be given to individual lateral asymmetries in running gait, especially during periods of increased training load and physiological stress typical of altitude training camps.

Taken together, these two aspects—acute hypoxia and potentially different running course conditions—may be relevant to training management, particularly at the beginning of a camp when no adaptation has yet taken place. It remains unclear how the aforementioned additional contributing factors to total physiological and biomechanical effort may further increase the load of a typical running session outside an athletics tartan track. This study aims to bridge this research gap through a real-world experimental approach to elucidate the effects of acute altitude-induced hypoxia and specific running surfaces on the biomechanical loading at relevant running speeds. We hypothesised that acute hypoxia might induce increases in CAD and PTA and a decrease in PTA asymmetry, and that natural surface conditions would elevate asymmetry.

## 2. Materials and Methods

### 2.1. Participants

Ten healthy, trained amateur triathletes and runners (4 females, 6 males) participated in this study. All subjects were adult native lowlanders and had been regularly living at an elevation between 50 and 160 m AMSL for at least 10 years. They exhibited no signs of cardiac, pulmonary or inflammatory diseases, nor did they report any orthopaedic issues at the time of the examinations. The subjects accomplished an average weekly running mileage of 29.5 ± 13.5 km and a regular exercise load of 10.7 ± 3.7 h per week. The performance-based inclusion criterion was a seasonal best of under 45 min over 10 km road running for the males (<4:30 min km^−1^) and under 60 min for the females (<6:00 min km^−1^). Detailed characteristics of the cohort are summarised in Table 1. Written informed consent was obtained from all participants prior to their participation. The study adhered to the Declaration of Helsinki and received approval from the Ethics Committee of the Department of Engineering and Industrial Design at the Magdeburg-Stendal University of Applied Sciences (approval numbers EKIWID-2023-09-001RM and EKIWID-2023-09-001RMII).

Due to the exploratory nature and multifactorial design, only an exemplary sample size estimation based on a single influencing factor and a single outcome variable was possible. Alda-Blanco et al. [40] reported a significant difference in HR of 5 bpm in trained runners when running on substantially different surfaces (tartan track vs. grass), with a pooled standard deviation of 8 bpm. Given the within-study design, we assumed a correlation of 0.75 between repeated measurements, resulting in a Cohen’s *d* of 0.92. To achieve a statistical power of 0.8 with a type I error level of *α* = 0.05, a minimum of 9 participants needed to be included in this study. Based thereon, we chose 11 subjects to allow for a dropout rate of up to 20%. Of those 11 participants, 10 eventually completed the study.

### 2.2. Study Design

**Figure 1:**
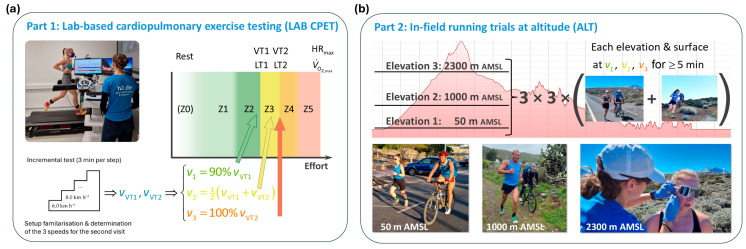
Study design. The study comprised two parts: (**a**) a laboratory-based cardiopulmonary exercise testing (LAB CPET) at virtual sea level, and (**b**) in-field running trials at altitude on the island of Tenerife (ALT, red elevation profile to scale). From the LAB CPET, the subjects’ individual ventilatory thresholds 1 and 2 (VT1 and VT2) were derived, and based thereon, the individual running speeds *v*_1_, *v*_2_ and *v*_3_ were defined. In the ALT sessions, these speeds were run with a slight intensity adjustment at elevations of approximately 50 m, 1000 m and 2300 m AMSL, both on road and trail surfaces; see text for details.

**Figure 2:**
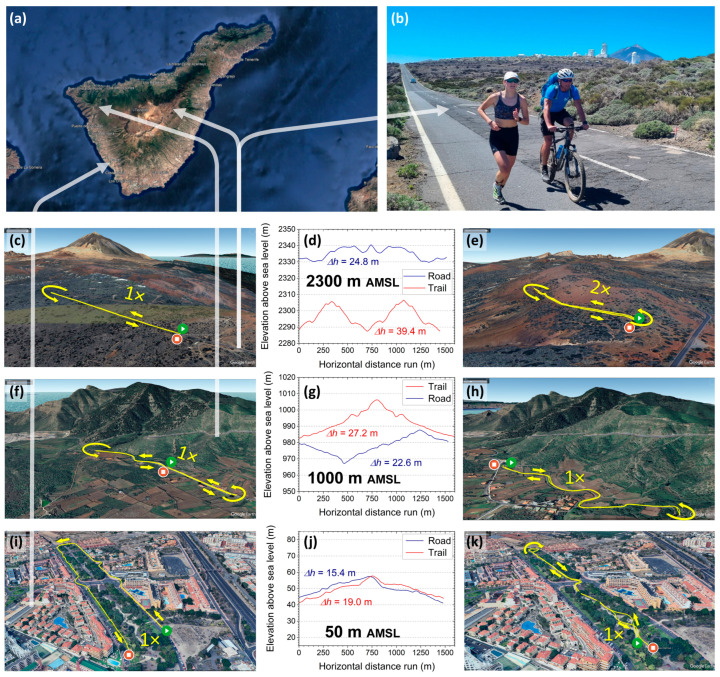
Running courses employed in this study. (**a**) Areas on the island of Tenerife, Spain, where the courses were situated. (**b**) Runner with accompanying cyclist for speed reference and IMU radio signal transmissions. (**c**–**k**) A 3 × 3 figure matrix depicting (left column) ROAD courses, (right column) TRAIL courses and (centre column) elevation profiles of the courses with total ascents Δh. See text for details. Parts of the topographic illustrations have been rendered using Google Earth Pro 7.3.6.10441 (Google LLC, Mountain View, CA, USA).

We employed a crossover design with counterbalanced surface conditions comprising two parts: (1) a laboratory-based cardiopulmonary exercise testing (LAB CPET) at virtual sea level (56 m AMSL, Figure 1a) and (2) a block of in-field running trials at three different altitude levels (ALTs) of approximately 50 m, 1000 m and 2300 m AMSL (Figure 1b).

#### 2.2.1. Part 1: Laboratory-Based Cardiopulmonary Exercise Testing (LAB CPET)

The LAB CPET was conducted in Magdeburg, Germany, between February and March 2024. First, participants’ physical health and suitability were confirmed through physical examination, self-reporting of health status, a questionnaire addressing physical activity and exercise patterns, and current performance metrics. Second, the subjects underwent a CPET on a motorised treadmill (Star Trac FreeRunner 10TRx, Core Health 6 Fitness, Vancouver, BC, Canada) using an established standardised incremental protocol [41,42], starting at a running speed of 6.0 km h^−1^ with 3 min stages and increments of 2.0 km h^−1^ until voluntary exhaustion. A 1% belt incline was used to compensate for the absence of air drag on the treadmill [43]. During each stage, the subjects’ ventilation, oxygen uptake and carbon dioxide output were continuously monitored through breath-by-breath spirometry (MetaMax 3B, CORTEX Biophysik GmbH, Leipzig, Germany) with appropriately sized face masks (7450 Series V2 Mask; Hans Rudolph, Inc., Shawnee, KS, USA). HR was continuously assessed using a multisport smartwatch (Garmin Forerunner 955 Solar, Garmin Ltd., Olathe, KS, USA) connected to a compatible chest strap (Garmin HRM Swim, Garmin Ltd., Olathe, KS, USA). HR measurements through this combination of wearables have recently been shown to be accurate within ±1 bpm by direct comparison to electrocardiography [44].

Based on those cardiopulmonary gas exchange data, the individual ventilatory thresholds 1 (VT1) and 2 (VT2) were derived by two independent, experienced researchers. Furthermore, LAC levels at volitional exhaustion (LAC_ex_) were measured by capillary blood sampling (Lactate SCOUT 4 Solo, EKF-diagnostic GmbH, Barleben, Germany), while RPE was assessed using the Borg 6–20 scale [45]. Based on the individual running speeds at VT1 (*v*_VT1_) and at VT2 (*v*_VT2_), three running speeds for in-field testing, i.e., *v*_1_, *v*_2_ and *v*_3_, were defined as

(1) $$v_{1}:=90\%\cdot v_{\mathrm{VT}1};\;\;\;\;v_{2}:=\frac{1}{2}\left(v_{\mathrm{VT}1}+v_{\mathrm{VT}2}\right);\;\;\;\;v_{3}:=\;100\%\cdot v_{\mathrm{VT}2}$$

for each subject individually. These speeds cover the typical range of paces used in distance running-specific training, i.e., Zone 2 “easy running” (within the framework of the five-zone model typically used in training practice [46]), Zone 3 “threshold training” and Zone 3→4 “threshold to ${\dot{V}}_{O_{2,\;max}}$” (Figure 2a) [41].

#### 2.2.2. Part 2: Altitude Trials (ALTs)

All ALTs were carried out on the island of Tenerife, Spain, within two weeks in March 2024 (Figure 2a). The participants spent most of their stay at or near sea level, except for the days of the ALTs (“sleep low, train high”). Before each trial, they were transferred to the altitude measurement sites by car within a travel time of ½ to 1 h. Measurements started within 1 h upon arrival at the specified elevation, ensuring an acute state of hypoxia.

At each of the three altitudes of 50, 1000 and 2300 m AMSL, the participants ran two predefined courses with different surfaces—road (ROAD, Figure 2c,f,i) and trail (TRAIL, Figure 2e,h,k)—at their individually set running speeds *v*_1_, *v*_2_ and *v*_3_. All courses were on public, paved roads with an asphalt surface in good condition (ROAD) or publicly accessible hiking paths with sufficiently even surfaces and similar ground conditions featuring a mixture of natural soil, gravel and volcanic ashes (TRAIL). All but one course consisted of a single lap and were approximately 1500 m long, implying that each subject had to run for at least 5 min to complete it. In one case (2300 m AMSL on TRAIL), the course consisted of two consecutive identical laps due to local topographical constraints. For each course, the start and end points were identical within ±20 m. The relative elevation profiles (Figure 2d,g,j), i.e., ascending and descending vertical metres and inclines, were chosen such that the expected climbing effort remained comparable between the courses given the local conditions and topological and geographical constraints, ranging between Δh = 15–25 m (ROAD) and 19–39 m (TRAIL), respectively. The upper boundary of the remaining biassing effect due to varying profiles was estimated to be less than 2.8% through the calculation of grade-adjusted paces [47]. Moreover, the experimental trials were scheduled in such a way that, across all measurement days, temperatures (20 ± 3 °C) and relative humidity (60 ± 10%) were relatively comparable at all elevations during the runs, taking advantage of the stable subtropical desert climate of the central and southern regions of Tenerife in March.

The order of uphill and downhill segments within a single course was dictated by the demands of practical feasibility, and its inevitable variability between courses was not deemed to possess the capacity to substantially alter the outcome in terms of time-averaged biomechanical variables. To facilitate the selection of the correct individual pace, a cyclist or an experienced, trained runner accompanied the participant at the specific target speed (Figure 2b), depending on the speed and local restrictions (particularly restrictions within the Teide National Park not allowing cyclists to use certain pathways). In addition to providing a reliable pacing reference, the accompanying person carried the IMU receiving station in their backpack (see Figure 2b and photo insets in Figure 1b at the top right, bottom left and centre).

In this way, in total, 3 elevations × 3 speeds × 2 surfaces = 18 ALT running bouts were conducted by each subject within a total time frame of 8 days. All six bouts belonging to the same elevation were completed as a combined trial on the same day. Between these elevation trials, at least two days of rest were granted, with only compensatory training.

Within an elevation trial, the order from *v*_1_ to *v*_2_ and *v*_3_ for a given surface was fixed to reduce the recovery time required between the bouts. Since at *v*_1_, by definition, LAC accumulation is negligible, the *v*_1_ runs were unlikely to bias the physiological baseline of the *v*_2_ runs, and, instead, they could serve as a standardised familiarisation routine for each course. Similarly, the potentially elevated LAC level at the end of the *v*_2_ run was not expected to significantly affect the experimental outcome at *v*_3_ because, according to the definition of VT2, a substantial lactate accumulation was anticipated to occur at that speed, marking the individual’s maximum LAC steady state. Therefore, both the *v*_2_ and the *v*_3_ runs could start shortly (i.e., 3–5 min) after the previous run at *v*_1_ or *v*_2_ had been completed. The remaining effect of LAC accumulation during *v*_2_ on the subsequent run at *v*_3_ can be regarded as negligible, as confirmed by the large numerical differences that were experimentally observed between the LAC levels after runs at *v*_2_ and *v*_3_. In contrast, the order of the two surfaces was randomised to minimise the effects of familiarisation with altitude. Blinding was not possible for obvious reasons of geographical clarity. Between the two surface trials at a given elevation, a compensatory recovery period of at least 15 min was granted until LAC levels in the subjects returned to roughly their baseline value, accounting for the generally substantial LAC accumulation in the *v*_3_ run of the previous surface trial.

Because altitude heightens physiological effort due to hypoxia, while the decrease in air pressure (e.g., a drop from approximately 1013 mbar at sea level to roughly 800 mbar at 2000 m AMSL) is considered important given the biomechanical objective of this study, the zone-based running speeds *v*_1_, *v*_2_ and *v*_3_ were slightly reduced at 1000 m and 2300 m AMSL to ensure the same relative effort in terms of percentage of maximum heart rate (%HR_max_) and, correlated with it, the Borg 6–20 scale for RPE [48,49]. Based on the experimental findings of Wehrlin et al. in trained endurance athletes, an increase in submaximal HR of +6.8 bpm (+5.1%) per 1000 m in elevation could be expected for efforts near VT1 (i.e., specifically, 55% ${\dot{V}}_{O_{2,\;max}})$, and a drop in maximal HR of −1.9 bpm (−1.0%) per 1000 m [50,51]. Hence, by combining these two findings, running at the same submaximal speed (in terms of km h^−1^) at higher altitude will require a heightened level of relative physiological effort (in terms of %HR_max_ or RPE), owing to both the higher submaximal HR and lower maximal HR. Numerically, the combination of the specific experimental results of Wehrlin et al. yields an approximate factor for the expected increase in %HRmax and RPE of $\left(1.0000+\frac{55.00\%\;{\dot{V}}_{O_{2,\max}}}{62.17\%{\dot{V}}_{O_{2,\max}}}\cdot 00.5113\right)\cdot \left(1.0000-0.0101\right)\approx 1.0347$, i.e., +3.5% per 1000 m in elevation. In this context it should be noted that other researchers have reported slightly higher reductions in maximal HR at altitude as summarised, e.g., by Lundby et al. [7], resulting in potentially slightly lower correction factors. A spot check with two subjects from our cohort yielded a correction factor of +3.3% per 1000 m, so that we chose to reduce the running speeds *v*_1_, *v*_2_ and *v*_3_ uniformly by −3.3% and −7.8% at 1000 m and 2300 m AMSL, respectively. Importantly, the adequacy of this adjustment, aiming at comparable relative physiological efforts at the three running speeds despite the effects of acute hypoxia, was later reconfirmed by the RPE results of the ALT runs of all subjects (see Section 3 below).

### 2.3. Biomechanical and Physiological Measurements During ALTs

Before the *v*_1_ runs, LAC levels of the subjects were assessed applying the same procedure and instrumentation as in LAB CPET. In addition, LAC was measured after each bout at *v*_1_, *v*_2_ and *v*_3_. During those consecutive capillary blood samplings, RPE was collected in an identical fashion as in the LAB CPET procedure. Similarly, during all runs, the athletes wore the same smartwatches and chest straps for HR measurements as in the LAB CPET condition. Furthermore, the subjects were equipped with three synchronised inertial measurement units (IMUs) of the type Xsens MTw Awinda (Movella Inc., Enschede, the Netherlands), two worn at their distal tibiae and one at their sacrum. The IMUs collected tri-axial accelerations (±16 *g*; 1 *g* = 9.80665 m s^−2^), angular velocities (±2000° s^−1^) and Euler angles at an internal data sampling rate of 1000 Hz and converted them to a time-continuous data stream of 120 Hz through strap-down integration and sensor fusion. These time series were transmitted to the receiving base station of the accompanying cyclist or runner using the 2.4 GHz band with a range of 50 m in free space. The employed IMUs have been widely used and validated in sport sciences and biomechanical research, particularly under in-field conditions [18,19,52].

### 2.4. Data Post Processing

All continuous HR readings of an individual running bout were averaged over its duration using the manufacturer’s software (Garmin Connect, Garmin Ltd., Olathe, KS, USA). The tri-axial acceleration time series of the two tibial sensors and the sacral sensor were analysed following established running gait segmentation algorithms based on magnitude peak detection described in previous studies using LabVIEW 2025 Q1 64-bit (National Instruments Corp., Austin, TX, USA) [18,19,42]. The resulting PTA values in terms of 3D vector magnitude were averaged over the duration of a running bout by discarding an initialisation and finalisation margin of 15 s each. Using these mean PTA of the left (*a*_L_) and right shank (*a*_R_), the lateral asymmetry index (LA) for a specific bout of an individual was calculated as [18]

(2) $$\mathrm{LA}=\frac{a_{L}-a_{R}}{\frac{1}{2}\left(a_{L}+a_{R}\right)};\;\;\;\;\;\mathrm{aLA}=\left|\mathrm{LA}\right|.$$

Because LA can take both signs, i.e., LA≥0 for higher PTA in the left leg and LA<0 for higher PTA in the right leg, summing over LA of different individuals might numerically cancel out effects of external factors, and thus conceal them. Therefore, the absolute lateral asymmetry index aLA, i.e., the absolute value of LA, is considered in all group means instead of LA. Moreover, residual shock at the pelvis (RSh) was derived from the mean peak tibial and mean peak sacral (*a*_Sac_) acceleration values through

(3) $$\mathrm{RSh}=\frac{a_{\mathrm{Sac}}}{\frac{1}{2}\left(a_{L}+a_{R}\right)}.$$

Residual shock generally describes the portion of combined impact and active peak shock during stance phase that is transferred by the musculoskeletal system from the distal caudal joints and body segments, such as the ankles, to the more cranial body parts, e.g., pelvis, trunk and head. On this route, it is continuously dampened by the natural shock attenuation mechanisms of the human body, e.g., eccentric joint flexion, energy absorption of muscles, tendons and cartilage, and, conditionally, also active compensation. In this study, we analysed only the pelvic aspect of residual shock as defined by RSh.

CAD was obtained as the frequency of the combined left and right leg (and sacral) impact acceleration peaks in the continuous acceleration time series [53].

### 2.5. Statistical Analysis

All data were processed using Microsoft Excel 2021 (Microsoft Corporation, Redmond, WA, USA) and analysed using IBM SPSS Statistics (Version 29; IBM, Armonk, New York, NY, USA). Unless otherwise stated, tables show mean values and standard deviations (SDs). The Shapiro–Wilk test was used to test for normality, and the Mauchly test for sphericity. Primary analysis was conducted using a three-way analysis of variance with repeated measures (rmANOVA) and post hoc tests adjusted for multiple comparisons using the Bonferroni correction. If sphericity could not be assumed, the Greenhouse-Geisser correction was applied. The level of significance was set to *p* < 0.05. Effect sizes in the rmANOVA were assessed based on partial eta squared (*η*_p_^2^) and rated according to Cohen: negligible for *η*_p_^2^ < 0.01, small for 0.01 ≤ *η*_p_^2^ < 0.06, medium for 0.06 ≤ *η*_p_^2^ < 0.14, and large for *η*_p_^2^ ≥ 0.14 [54]. Analogously, effect sizes in the (Bonferroni-corrected post hoc) *t*-test comparisons were calculated and evaluated according to Cohen’s *d* with negligible (*d* < 0.2), small (0.2 ≤ *d* < 0.5), medium (0.5 ≤ *d* < 0.8) and large effects (*d* ≥ 0.8) [54].

## 3. Results

During the LAB CPET incremental treadmill test, the participants achieved a mean maximum running speed of 17.0 ± 1.7 km h^−1^ with an average relative oxygen uptake per body mass of ${\dot{V}}_{O_{2},\mathrm{peak}}$ = 54.9 ± 5.9 mL kg^−1^ min^−1^. Table 2 summarises further outcome variables and derived performance metrics of the LAB CPET.

**Figure 3:**
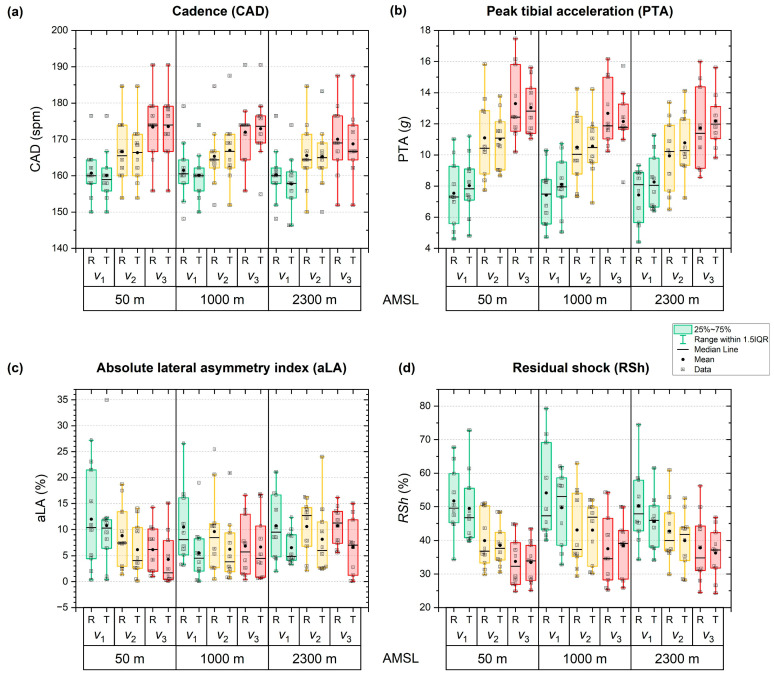
Biomechanical outcome metrics at the end of the running bouts as a function of elevation, running speed and surface. (**a**) Cadence, (**b**) Peak tibial acceleration, (**c**) Absolute asymmetry index, (**d**) Residual shock. The running speeds *v*_1_ (green), *v*_2_ (yellow) and *v*_3_ (red) were defined on an individual basis according to the ventilatory thresholds 1 and 2 (see Equation (1) in text). Surface conditions were road (R) and trail (T).

Summarising the results of all ALT runs are presented in terms of detailed numerical data and test statistics in Table A1 in Appendix A1. The corresponding post hoc test results for the three-way rmANOVA are provided in Table A2 there. Figure 3 depicts the cohort’s mean for CAD, PTA, aLA and RSh as a function of altitude, running speed and running surface. Running speed had the most significant impact on all biomechanical metrics. There were also minor interactions noted between speed and elevation, as well as between speed and surface. However, the effect of elevation was only apparent in cadence. Meanwhile, surface conditions did not affect the biomechanical parameters.

The corresponding results for the control variables HR, LAC and RPE are shown and discussed in Appendix A2, essentially confirming that the perceived physiological effort in terms of RPE was practically identical between elevations for fixed other conditions within measurement uncertainty. The mean relative difference between the planned and the actually run speeds, as determined by GPS/GNSS tracking, was −1.1 ± 1.6% at *v*_1_ (i.e., the subjects actually ran slightly faster), +0.3 ± 2.0% (slightly more slowly) at *v*_2_ and +1.4 ± 2.7% at *v*_3_ (slightly more slowly).

Concerning the biomechanical metrics, CAD (Figure 3a) was unaffected by surface condition (*p* = 0.610; Table A1), whereas elevation (*p* < 0.001; *η*_p_^2^ = 0.659) and running speed (*p* < 0.001; *η*_p_^2^ = 0.894) exhibited both significant and strong effects (Table A1). Elevation-related changes were small, with only a slight but significant decrease from sea level to 2300 m AMSL (167 ± 8 vs. 165 ± 9 spm; *p* < 0.006). Running speed elicited robust increases in cadence across all levels (*v*_1_: 160 ± 7 spm, *v*_2_: 166 ± 9 spm, *v*_3_: 172 ± 9 spm; all *p* < 0.001) Table A2). Significant speed-elevation interactions revealed lower cadences at higher elevations, particularly at *v*_1_ (2300 vs. 1000 m AMSL) and *v*_3_ (1000 and 2300 vs. 50 m AMSL), with moderate-to-large effect sizes (Cohen’s *d* = −0.79 to −1.44; *p* < 0.034). Speed–surface interactions were minimal, with only one significant effect at *v*_2_ and 1000 m AMSL, cadence was moderately higher on TRAIL than ROAD (*p* < 0.07). All other interaction effects were non-significant.

PTA (Figure 3b) was largely affected by the main factor running speed (*p* < 0.001, *η*_p_^2^ = 0.926; Table A1), but neither by elevation (*p* = 0.197) nor surface (*p* = 0.614) alone. However, there were significant interaction effects between running speed and elevation (*p* < 0.001, *η*_p_^2^ = 0.503) and running speed and surface (*p* = 0.003, *η*_p_^2^ = 0.477). Other interactions were not significant (Table A1). Specifically, PTA was 7.8 ± 1.6 *g* at *v*_1_, 10.6 ± 1.8 *g* at *v*_2_ and 12.5 ± 1.6 *g* at *v*_3_, with all differences being highly significant (*p* < 0.001; Table A2). Comparing surface results, mean PTA, averaged across all elevations and running speeds, was similar on ROAD (10.2 ± 2.1 *g*) and TRAIL (10.5 ± 1.4 *g*; Table A2). Similarly, PTA was comparable between elevations, amounting to 10.7 ± 1.9, 10.2 ± 1.5 and 10.1 ± 1.6 *g* at 50, 1000 and 2300 m AMSL, respectively (Table A2). Interaction analyses indicated that elevation-related reductions in PTA occurred only at higher running speeds and exclusively on ROAD. Specifically, PTA was lower at *v*_2_ at 1000 m (−5.4%) and 2300 m AMSL (−10.4%) compared with 50 m AMSL, and at *v*_3_ at 2300 m AMSL (−11.9%), with moderate to large effect sizes (Cohen’s *d* = −0.73 to −1.11; *p* ≤ 0.048). No further significant interaction effects were observed.

Considering aLA (Figure 3c), none of the factors elevation (*p* = 0.664), running speed (*p* = 0.058) or surface (*p* = 0.150) exhibited a significant influence on running gait symmetry (Table A1). There was a trend of smaller asymmetries at higher speeds, with mean absolute asymmetries of 9.3 ± 4.6%, 8.2 ± 2.7% and 6.9 ± 2.3% at *v*_1_, *v*_2_ and *v*_3_, respectively, although not reaching significance (Table A1 and Table A2). Mean values were comparable across elevations (50 m: 8.0 ± 4.6%, 1000 m: 7.5 ± 4.7%, 2300 m AMSL: 8.9 ± 2.0%; all *p* = 1.000) and did not differ significantly between ROAD and TRAIL (9.5 ± 4.9% vs. 6.8 ± 3.0%; *p* = 0.150; Table A2).

Similarly to PTA, RSh (Figure 3d) was strongly affected by running speed (*p* < 0.001; *η*_p_^2^ = 0.897) and the interaction between speed and elevation (*p* < 0.001; *η*_p_^2^ = 0.425), while the other main effects (*p* > 0.218) and interactions (*p* > 0.181) demonstrated no significant effect (Table A1). Particularly, increasing the speed significantly reduced RSh from 50.1 ± 8.0% at *v*_1_ to 41.2 ± 7.4% at *v*_2_ and 36.2 ± 7.6% at *v*_3_ (all pairwise post hoc comparisons with *p* < 0.001; Table A2). RSh was similar across elevations (41.1–44.3%) and between surface conditions (ROAD: 43.4 ± 9.1% vs. TRAIL: 41.6 ± 7.1%; Table A2), with no significant difference therein. Interaction analysis revealed significant elevation-related increases in RSh only at *v*_3_ on TRAIL, with higher values at 1000 m (+14.8%) and 2300 m AMSL (+8.4%) compared to sea level (*p* < 0.032). All other interaction effects were non-significant.

## 4. Discussion

The aim of this study was to systematically elucidate how acute hypoxia and surfaces specific to altitude training camps affect the biomechanical loading at the range of running speeds relevant to structured training in long-distance running. It was hypothesised that acute hypoxia may induce increases in CAD and PTA and a decrease in PTA asymmetry. No hypotheses as to the spatiotemporal gait structure and the associated biomechanical loading in terms of CAD, PTA and between-leg lateral asymmetry were possible, leaving this aspect open to direct exploration in this study.

First, in terms of perceived exertion, acute altitude did not exhibit a statistically significant effect on RPE, confirming that the adjustment of running speeds at altitude conditions (i.e., −3.3% and −7.8% at 1000 and 2300 m AMSL, respectively; see above) as derived from the literature and a prior spot check was indeed successful in inducing a comparable subjective physiological effort at different elevations and thus oxygen partial pressures. In view of this, it seems reasonable to postulate that reducing running speeds by roughly −3% per +1000 m in elevation gain may compensate for the—subjectively—higher physiological effort at altitude. For a running pace of, e.g., 4:15 min km^−1^, this would imply a reduction in pace to 4:30 min km^−1^ at 2000 m AMSL, thus approximately +15 s per kilometre, which is in good quantitative agreement with current coaching procedures. Moreover, in terms of the effect of surface, the study outcome suggests that the athletes did not perceive trail or road conditions as a particularly strenuous factor for the submaximal running speeds given.

Second, regarding the spatiotemporal structure of running gait, running speed was found to strongly affect cadence, with an average increase of +2.2 spm per +1 km h^−1^, which is in formidable numerical accordance with the recent literature [53,55]. Statistically, elevation also exerted a strong effect on cadence, but the drop in cadence by −2.2 [−3.7, −0.7] spm from sea level (166.8 spm) to 2300 m AMSL (164.6 spm), i.e., −1.0 spm per +1000 m in elevation, was numerically small (−1.3%; Cohen’s *d* = −0.22) and fell within the margin of experimental uncertainty due to the speed adjustment and the practically inevitable small differences in total vertical metres between the courses. The observed slight numerical differences in cadence at different elevations at fixed speed and surface conditions are thus likely to possess limited practical relevance. Moreover, surface did not substantially affect step frequency, neither as a main factor nor in interactions, yielding practically equivalent results in road running and trail running for a given speed and elevation. This suggests that the potentially decreasing effect of a more compliant surface on cadence (even asphalt vs. even turf), as recently reported by Moon et al. [21], may be cancelled out in trail running practice by an increase in PTA due to the inherent bumpier surface properties. Therefore, in view of our results, it can be concluded that the effect of uneven surface conditions on cadence is likely to play as decisive a role as mechanical surface compliance, but in the opposite direction.

As for biomechanical loading metrics, PTA demonstrated a strong dependence on running speed, with an average increase of +0.9 *g* per +1 km h^−1^, showing excellent numerical agreement with previous, laboratory-based studies [18]. Remarkably, neither elevation nor surface exerted significant main effects on PTA. However, there was a stable trend in road running of slightly to moderately lower PTA at higher elevations when combined with higher running speeds, which became significant for 1000 m and 2300 m vs. 50 m AMSL at *v*_2_ and for 2300 m vs. 50 m AMSL at *v*_3_. Although effect sizes and relative changes (−5%, −10% and −12%) remained within moderate ranges, this finding suggests that acute hypoxia does indeed induce some subtle changes in running gait structure on asphalt roads towards a potentially lower associated musculoskeletal loading at vigorous running speeds beyond the first ventilatory threshold. Given that this effect was observed only on asphalt and not in trail running, the underlying mechanisms are currently speculative. Possibly, a slight, subconscious adaptation in running gait may take place under acute hypoxia in order to match a new metabolic optimum under altered external conditions, perhaps similar to an effect that has been reported for hypogravity [42]. These changes may perhaps stay concealed in trail running because of the less stable spatiotemporal gait structure there. While this is highly speculative, it is evident that the observed phenomenon of lower PTA at altitude on ROAD at faster running speeds is unlikely to be a methodological artefact caused by the speed adjustments at altitude: Firstly, those adjustments (−3.3% at 1000 m AMSL and −7.8% at 2300 m; see above) were only approximately half the size of the observed drops in PTA. Secondly, the effect of lower PTA at altitude was absent at *v*_1_ on ROAD and, remarkably, also at all speeds on TRAIL. Furthermore, the numerically negligible trend towards lower cadences at altitude, particularly on ROAD, does not possess the effect size to explain the observed reductions in PTA either. Irrespective of this, the experimental outcome that road running and trail running result in virtually identical PTA metrics at a given running speed and elevation challenges the common presumption that running on trails may impose lower impact loading than on asphalt. Nonetheless, this outcome of the present study is in line with previous research that found that running speed is substantially more decisive than surface [18], and that asphalt, grass and tartan tracks induce practically the same PTA for a given running speed [18,21]. In terms of practical implications for altitude training camps, this finding suggests that some of the typical exercising convictions may be worth revisiting, given that well-runnable roads may be more easily accessible than similarly even and gentle trails.

With regard to the absolute lateral asymmetry index, all results across running speeds, elevations and surface conditions, stayed within the boundaries considered to be physiological in the pertinent literature [18]. Intriguingly, among all biomechanical and physiological measures investigated in this study, aLA was the only metric that remained fully unaffected by running speed, elevation and surface in terms of statistical significance. Despite not reaching statistical significance (*p* = 0.058), a tendency towards smaller absolute asymmetries with increasing speed was yet observable, a trend known from previous studies in well trained runners and trained amateur triathletes [18,24]. As to its explanation, it may be speculated that with increasing neuromuscular effort, the between-leg variance tends to reduce in order to optimise efficiency and avoid premature unilateral muscular fatigue. Nonetheless, the fact that aLA was essentially not altered to any substantial extent by the external factors in this study supports the notion that lateral asymmetry may be a rather stable intrinsic feature of individual running gait, at least for a given running speed and footwear condition [18,19,24]. In other words, focussing on practical relevance, this outcome implies that neither acute hypoxia nor changes in running surface during an altitude training camp should be a cause for concern with regard to a potential deterioration of existing between-leg loading asymmetries.

Finally, residual shock at the pelvis exhibited a strong reduction by running speed throughout elevations and surfaces, suggesting that at faster speeds, the internal attenuation of tibial impact shock by the ankle, knee and hip joints substantially increases in magnitude. This finding is in line with previous laboratory-based studies, as was the range of RSh values observed in this study [18,24]. Remarkably, RSh was practically the same in road and trail running, another finding that has the potential to question the common belief of purportedly less impact loading on natural surfaces compared to paved asphalt roads. Moreover, acute hypoxia did not substantially impact shock attenuation, with the only, rather moderate increases in RSh observed in trail running at the fastest speed at sea level vs. 1000 m and 2300 m AMSL, respectively. While it could be speculated, somewhat in line with the hypothesis that PTA might be slightly reduced at higher speeds at altitude, that a slightly higher physiological demand of running under acute hypoxia might have a diminishing effect on non-essential, comfort-related features of running gait, the effect sizes of this observation are too small to warrant any practically meaningful interpretation.

From a practical training perspective, it remains important for coaches and athletes to note that running speed is one of the key factors for biomechanical changes, regardless of altitude conditions or different surfaces. Both cadence and PTA are strongly influenced by this, which can lead to increased biomechanical stress—and the risk of overuse injuries. This relation is substantially more important than the influence of altitude or surface. The absolute lateral asymmetry index seems to be less sensitive to external factors, as it remains practically unaffected by running speed, elevation and surface. The fact that virtually identical results for residual shock at the pelvis were obtained in asphalt and trail running should alert coaches that a supposedly “lower impact loading” of trail running seems questionable.

This study is subject to several limitations that must be accounted for. First, the relatively small sample size of 10 athletes results in a limited statistical power, increasing the likelihood of a type II error. Additionally, the sample size calculation was based on heart rate. Thus, for the biomechanical metrics, only a post hoc power analysis could be performed. Overall, the results related to surface showed no significant effects. This outcome is further supported by the post hoc power of the main effect of surface: CAD (power 1−β=0.065), PTA (0.076), RSh (0.120) and aLA (0.300). Due to the time constraints of the measurement trials, recovery phases between runs, particularly after runs at *v*_3_, may have been, in part, too short to allow for a full recovery back to baseline physiology in terms of HR and LAC. However, the randomisation of surface conditions among the subjects is thought to have minimised this bias, whereas randomising the running speeds for a fixed elevation and surface was not possible given the prolonged recovery times after *v*_3_. In addition, despite being similar, the topographical profiles of the running courses were neither identical between surface conditions nor between elevations, which may have impacted the biomechanical outcome to some—from our perspective still acceptable—extent. Finally, the slight adjustment of running speeds to yield akin ratings of perceived exertion at different elevations for a given running speed and surface helped to keep physiological loading comparable and ensure feasibility for all subjects. However, this adjustment might have slightly biased the exact quantitative interpretation of changes in the biomechanical metrics CAD, PTA and RSh. Nevertheless, the fact that the observed effects, if present and practically relevant, had a substantially higher magnitude than that of the speed adjustments supports our conception that this adjustment was a reasonable, and probably inevitable, trade-off between study objectives and study feasibility. In an ideal scenario, the ventilatory threshold of each subject would have been determined at each elevation using the same standardised treadmill setup. In practice, however, this is not feasible due to apparent technical and logistic constraints.

## 5. Conclusions

In conclusion, running speed seems to be the practically dominant factor defining biomechanical loading under acute hypoxia and varying surface conditions. In particular, the effect of acute altitude on PTA was found to be small and likely below the threshold of practical meaningfulness with respect to load measurement and injury prevention. In addition, trail running did not reduce PTA compared to running on asphalt surfaces, which may challenge some common beliefs among coaches. Moreover, between-leg asymmetry in running gait was not altered by elevation, surface or running speed. Shock attenuation also remained practically unaffected by elevation and surface conditions, but exhibited a positive association with running speed. In essence, coaches should prioritise controlling running speed rather than surface selection when managing biomechanical loading at altitude to optimise training and minimise injury risks.

## Figures and Tables

**Table 1:** General anthropometry and training characteristics of the study participants (*n* = 10).

Characteristic	Mean ± SD	Range
Age (years)	28.2 ± 9.2	18–44
Stature (cm)	176.0 ± 7.8	162.0–187.0
Body mass (kg)	68.2 ± 8.1	58.0–82.3
BMI (kg m^−2^)	22.1 ± 2.4	17.8–25.1
Weekly exercise load (hours per week)	10.7 ± 3.7	5.0–16.0
Weekly running mileage (km)	29.5 ± 13.5	10.0–50.0
Personal best 10 km road running (mm:ss)	42:36 ± 06:12	33:26–57:00

Abbreviations: BMI—body mass index. All values are given as means ± standard deviation.

**Table 2:** LAB CPET results of the study participants (*n* = 10).

Performance Outcome	Mean ± SD	Range	$\%\;{\dot{V}}_{O_{2,peak}}$
Achieved maximal running speed (km h^−1^)	17.0 ± 1.7	14.3–20.0	100.0 ± 0.0
HR_peak_ (bpm)	190 ± 6.3	178–200	100.0 ± 0.0
VE_peak_ (L min^−1^)	132 ± 24	101.2–177.3	100.0 ± 0.0
${\dot{V}}_{O_{2,\;\;peak}}\;$(mL min^−1^ kg^−1^)	54.9 ± 5.9	47–66	100.0 ± 0.0
LAC_ex_ (mmol L^−1^)	10.4 ± 3.6	6.0–17.3	–
RPE (Borg 20)	20 ± 0	20–20	100.0 ± 0.0
*v*_VT1_ (km h^−1^)	10.4 ± 0.7	9.4–12.0	69.1 ± 8.9
*v*_VT2_ (km h^−1^)	15.0 ± 1.6	12.3–17.8	93.9 ± 3.8
*v*_1_ (km h^−1^)	9.4 ± 0.8	8.5–11.3	62.2 ± 8.0
*v*_2_ (km h^−1^)	12.7 ± 1.2	11.3–15.2	81.5 ± 6.4
*v*_3_ (km h^−1^)	14.9 ± 1.9	12.2–17.8	93.9 ± 3.8

Abbreviations: HR_peak_: peak hear rate in beats per minute (bpm); VE_peak_: peak ventilation; ${\dot{V}}_{O_{2,peak}}$: peak oxygen uptake per time normalised to body mass, % ${\dot{V}}_{O_{2,\;peak}}$ relative percentage thereof; LAC_ex_ blood lactate concentration at volitional exhaustion; RPE: rating of perceived exertion based on the Borg 6–20 scale; *v*_VT1_: running speed at first ventilatory threshold; *v*_VT1_: running speed at second ventilatory threshold; *v*_1_, *v*_2_, *v*_3_ as defined in Equation (1).

**Figure A1:**
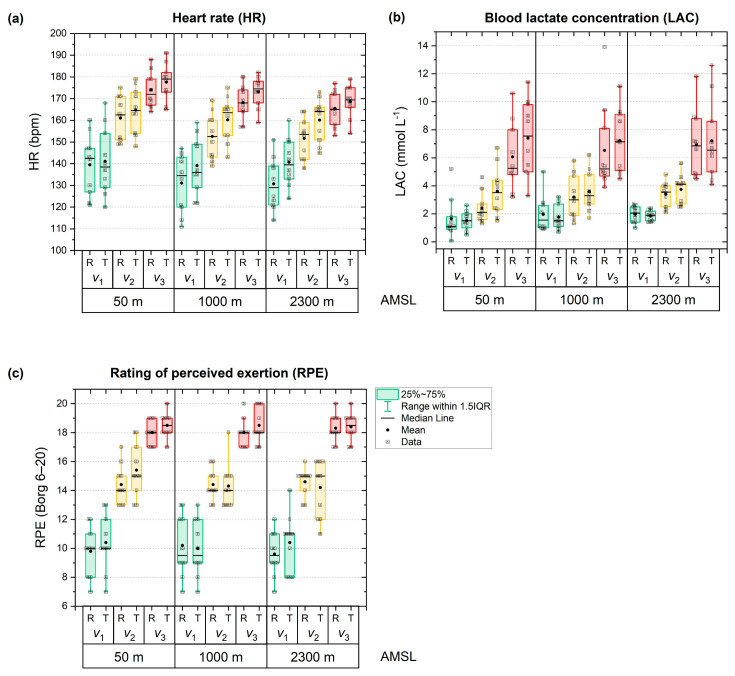
Physiological control variables at the end of the running bouts as a function of elevation, running speed and surface. (**a**) Heart rate, (**b**) blood lactate concentration, (**c**) rating of perceived exertion. The altitudes of 50, 1000 and 2300 m are given in terms of metres above mean sea level. All symbols and colour codes are as in Figure 3.

## Data Availability

The raw data for the results presented in this manuscript are available from the corresponding author upon reasonable request.

## Abbreviations

The following abbreviations are used in this manuscript:

%HR_max_	Percentage of maximum heart rate
aLA	Absolute lateral asymmetry index
ALT	In-field running trial at altitude
AMSL	Above mean sea level
bpm	Beats per minute
CAD	Cadence (step frequency)
HR	Heart rate
IMU	Inertial measurement unit
LA	Lateral asymmetry index
LAB CPET	Laboratory-based cardiopulmonary exercise testing
LAC	Blood lactate
LAC_ex_	Blood lactate at volitional exhaustion
PTA	Peak tibial acceleration
rmANOVA	Repeated measures analysis of variance
ROAD	Road surface
RPE	Rating of perceived exertion (RPE)
RSh	Residual shock
SD	Standard deviation
spm	Steps per minute
TRAIL	Trail surface
VE_peak_	Peak ventilation
${\dot{V}}_{O_{2,max}}$	Maximum oxygen uptake per unit time and body mass
${\dot{V}}_{O_{2,peak}}$	Peak oxygen uptake per unit time and body mass
VT1	Ventilatory threshold 1
VT2	Ventilatory threshold 2

## Appendix A

### Appendix A.1. Detailed Results Tables

**Table A1:** Biomechanical load metrics at end of runs along with the corresponding main effects of a three-way rmANOVA.

Load Metric	Elevation AMSL	Speed	Surface		Significance Level *p* and Effect Size *η*_p_^2^						
			ROAD	TRAIL	Elevation	Speed	Surface	Elevation × Surface	Elevation × Speed	Speed × Surface	Elevation × Speed × Surface
Cadence (CAD, spm)	50 m	*v*_1_	161 ± 7	160 ± 7	***p*** **< 0.001**	***p*** **< 0.001**	*p* = 0.610	*p* = 0.287	***p*** **< 0.001**	***p*** **= 0.003**	*p* = 0.135
		*v*_2_	167 ± 9	166 ± 9							
		*v*_3_	173 ± 9	173 ± 9							
	1000 m	*v*_1_	161 ± 8	160 ± 7							
		*v*_2_	165 ± 9	167 ± 9							
		*v*_3_	172 ± 9	173 ± 9							
	2300 m	*v*_1_	160 ± 8	158 ± 8	***η*****_p_****^2^** **= 0.659**	***η*****_p_****^2^** **= 0.894**	*η*_p_^2^ = 0.030	*η*_p_^2^ = 0.130	***η*****_p_****^2^** **= 0.487**	***η*****_p_****^2^** **= 0.481**	*η*_p_^2^ = 0.173
		*v*_2_	166 ± 10	165 ± 9							
		*v*_3_	170 ± 10	169 ± 9							
Peak tibial acceleration (PTA, g)	50 m	*v*_1_	7.5 ± 2.1	8.0 ± 1.9	*p* = 0.197	***p*** **< 0.001**	*p* = 0.614	*p* = 0.445	***p*** **< 0.001**	***p*** **= 0.003**	*p* = 0.422
		*v*_2_	11.1 ± 2.6	11.0 ± 1.8							
		*v*_3_	13.3 ± 2.4	13.0 ± 1.7							
	1000 m	*v*_1_	7.4 ± 1.9	8.1 ± 1.8							
		*v*_2_	10.5 ± 2.3	10.6 ± 2.0							
		*v*_3_	12.7 ± 2.1	12.2 ± 2.0							
	2300 m	*v*_1_	7.4 ± 1.8	8.3 ± 1.8	*η*_p_^2^ = 0.165	***η*****_p_****^2^** **= 0.926**	*η*_p_^2^ = 0.029	*η*_p_^2^ = 0.086	***η*****_p_****^2^** **= 0.503**	***η*****_p_****^2^** **= 0.477**	*η*_p_^2^ = 0.100
		*v*_2_	9.9 ± 2.3	10.8 ± 2.1							
		*v*_3_	11.7 ± 2.6	12.2 ± 1.7							
Lateral asymmetry index (LA, %)	50 m	*v*_1_	12.0 ± 9.4	10.8 ± 9.6	*p* = 0.664	*p* = 0.058	*p* = 0.150	*p* = 0.302	*p* = 0.089	*p* = 0.743	*p* = 0.389
		*v*_2_	8.8 ± 6.1	6.1 ± 5.3							
		*v*_3_	6.1 ± 4.7	4.3 ± 5.2							
	1000 m	*v*_1_	10.5 ± 7.4	5.5 ± 5.6							
		*v*_2_	9.6 ± 8.1	6.2 ± 6.3							
		*v*_3_	6.8 ± 5.9	6.7 ± 6.2							
	2300 m	*v*_1_	10.7 ± 6.3	6.5 ± 3.2	*η*_p_^2^ = 0.045	*η*_p_^2^ = 0.317	*η*_p_^2^ = 0.215	*η*_p_^2^ = 0.033	*η*_p_^2^ = 0.228	*η*_p_^2^ = 0.032	*η*_p_^2^ = 0.106
		*v*_2_	10.6 ± 5.2	8.1 ± 6.9							
		*v*_3_	10.7 ± 3.7	6.6 ± 5.5							
Residual shock (RSh, %)	50 m	*v*_1_	52 ± 10	49 ± 11	*p* = 0.218	***p*** **< 0.001**	*p* = 0.416	*p* = 0.775	***p*** **< 0.001**	*p* = 0.181	*p* = 0.537
		*v*_2_	40 ± 8	39 ± 6							
		*v*_3_	34 ± 8	33 ± 6							
	1000 m	*v*_1_	54 ± 14	50 ± 11							
		*v*_2_	43 ± 12	43 ± 9							
		*v*_3_	38 ± 11	38 ± 9							
	2300 m	*v*_1_	50 ± 11	46 ± 8	*η*_p_^2^ = 0.156	***η*****_p_****^2^** **= 0.897**	*η*_p_^2^ = 0.075	*η*_p_^2^ = 0.028	***η*****_p_****^2^** **= 0.425**	*η*_p_^2^ = 0.185	*η*_p_^2^ = 0.068
		*v*_2_	43 ± 9	40 ± 8							
		*v*_3_	38 ± 10	36 ± 8							

Comments: The cohort size was *n* = 10 participants. All outcome values are presented as means ± standard deviations. Significance levels *p* and effect sizes η_p_^2^ are based on a three-way repeated-measures analysis of variance (rmANOVA; see text). Significant results are highlighted in boldface.

**Table A2:** Post hoc comparisons for the biomechanical load metrics at the end of runs (three-way rmANOVA).

Load Metric	Factor	Post Hoc Testing	Mean Difference (95% CI)	Significance Level
Cadence (CAD, spm)	Elevation	50 vs. 1000 m AMSL	+0.3 (−0.5 to +1.1)	*p* = 0.849
		**50 vs. 2300 m AMSL**	**+2.2 (+0.7 to +3.7)**	***p*** **= 0.006**
		1000 vs. 2300 m AMSL	+1.9 (+0.7 to +3.0)	*p* = 0.383
	Speed	***v*****_1_** **vs. *v*_2_**	**−5.9 (−8.5 to −3.3)**	***p*** **< 0.001**
		***v*****_1_** **vs. *v*_3_**	**−11.7 (−15 to −8.1)**	***p*** **< 0.001**
		***v*****_2_** **vs. *v*_3_**	**−5.7 (−7.7 to −3.8)**	***p*** **< 0.001**
	Surface	Road vs. Trail	+0.4 (−1.4 to +2.2)	*p* = 0.610
Peak tibial acceleration (PTA, g)	Elevation	50 vs. 1000 m AMSL	+0.43 (−0.46 to +1.32)	*p* = 0.563
		50 vs. 2300 m AMSL	+0.62 (−0.37 to +1.61)	*p* = 0.303
		1000 vs. 2300 m AMSL	+0.18 (−0.88 to +1.25)	*p* = 1.000
	Speed	***v*****_1_** **vs. *v*_2_**	**−2.85 (−3.49 to −2.20)**	***p*** **< 0.001**
		***v*****_1_** **vs. *v*_3_**	**−4.71 (−5.96 to −3.46)**	***p*** **< 0.001**
		***v*****_2_** **vs. *v*_3_**	**−1.86 (−2.64 to −1.08)**	***p*** **< 0.001**
	Surface	Road vs. Trail	−0.28 (−1.48 to +0.92)	*p* = 0.614
Lateral asymmetry index (LA, %)	Elevation	50 vs. 1000 m AMSL	+0.5 (−3.2 to +4.2)	*p* = 1.000
		50 vs. 2300 m AMSL	−0.9 (−5.7 to +4.0)	*p* = 1.000
		1000 vs. 2300 m AMSL	−1.3 (−5.6 to +2.9)	*p* = 1.000
	Speed	*v*_1_ vs. *v*_2_	+1.1 (−1.2 to +3.4)	*p* = 0.608
		*v*_1_ vs. *v*_3_	+2.5 (−0.8 to +5.7)	*p* = 0.166
		*v*_2_ vs. *v*_3_	+1.4 (−0.3 to +3.0)	*p* = 0.106
	Surface	Road vs. Trail	+2.8 (−1.2 to +6.8)	*p* = 0.150
Residual shock (RSh, %)	Elevation	50 vs. 1000 m AMSL	−3.2 (−8.5 to +2.1)	*p* = 0.343
		50 vs. 2300 m AMSL	−1.0 (−4.8 to +2.9)	*p* = 1.000
		1000 vs. 2300 m AMSL	+2.2 (−4.0 to +8.4)	*p* = 0.979
	Speed	***v*****_1_** **vs. *v*_2_**	**+8.9 (+6.2 to +11.7)**	***p*** **< 0.001**
		***v*****_1_** **vs. *v*_3_**	**13.9 (+9.5 to +18.4)**	***p*** **< 0.001**
		***v*****_2_** **vs. *v*_3_**	**+5.0 (+2.6 to +7.4)**	***p*** **< 0.001**
	Surface	Road vs. Trail	+1.8 (−3.0 to +6.6)	*p* = 0.416

Comments: The cohort size was *n* = 10 participants. Significance levels *p* and confidence intervals CI were derived from Bonferroni-corrected post hoc testing for the three-way repeated-measures analysis of variance (rmANOVA; see text). Significant results are highlighted in boldface.

**Table A3:** Post-hoc comparisons of significant interaction effects for the biomechanical load metrics (three-way rmANOVA). Significant results are highlighted in boldface.

Load Metric	Factor	Post Hoc Testing	Mean Difference (95% CI)	Significance Level
Cadence (CAD, spm)	Elevation × Speed	**50 m × *v*_1_ vs. 50 m × *v*_2_**	**−6.090**	**<0.001**
		**50 m × *v*_1_ vs. 50 m × *v_3_***	**−13.090**	**<0.001**
		50 m × *v*_1_ vs. 1000 m × *v_1_*	−0.430	0.317
		**50 m × *v*_1_ vs. 1000 m × *v_2_***	**−5.770**	**<0.001**
		**50 m × *v*_1_ vs. 1000 m × *v_3_***	**−12.080**	**<0.001**
		**50 m × *v*_1_ vs. 2300 m × *v_1_***	**1.380**	**0.036**
		**50 m × *v*_1_ vs. 2300 m × *v_2_***	**−5.030**	**<0.001**
		**50 m × *v*_1_ vs. 2300 m × *v_3_***	**−9.030**	**<0.001**
		**50 m × *v*_2_ vs. 50 m × *v_3_***	**−7.000**	**<0.001**
		**50 m × *v*_2_ vs. 1000 m × *v_1_***	**5.660**	**<0.001**
		50 m × *v*_2_ vs. 1000 m × *v_2_*	0.320	0.57
		**50 m × *v*_2_ vs. 1000 m × *v_3_***	**−5.990**	**<0.001**
		**50 m × *v*_2_ vs. 2300 m × *v_1_***	**7.470**	**<0.001**
		50 m × *v*_2_ vs. 2300 m × *v_2_*	1.060	0.113
		**50 m × *v*_2_ vs. 2300 m × *v_3_***	**−2.940**	**0.012**
		**50 m × *v*_3_ vs. 1000 m × *v_1_***	**12.660**	**<0.001**
		**50 m × *v*_3_ vs. 1000 m × *v_2_***	**7.320**	**<0.001**
		**50 m × *v*_3_ vs. 1000 m × *v_3_***	**1.010**	**0.016**
		**50 m × *v*_3_ vs. 2300 m × *v_1_***	**14.470**	**<0.001**
		**50 m × *v*_3_ vs. 2300 m × *v_2_***	**8.060**	**<0.001**
		**50 m × *v*_3_ vs. 2300 m × *v_3_***	**4.060**	**<0.001**
		**1000 m × *v*_1_ vs. 1000 m × *v_2_***	**−5.340**	**<0.001**
		**1000 m × *v*_1_ vs. 1000 m × *v_3_***	**−11.650**	**<0.001**
		**1000 m × *v*_1_ vs. 2300 m × *v_1_***	**1.810**	**0.003**
		**1000 m × *v*_1_ vs. 2300 m × *v_2_***	**−4.600**	**<0.001**
		**1000 m × *v*_1_ vs. 2300 m × *v_3_***	**−8.600**	**<0.001**
		**1000 m × *v*_2_ vs. 1000 m × *v_3_***	**−6.310**	**<0.001**
		**1000 m × *v*_2_ vs. 2300 m × *v_1_***	**7.150**	**<0.001**
		1000 m × *v*_2_ vs. 2300 m × *v_2_*	0.740	0.089
		**1000 m × *v*_2_ vs. 2300 m × *v_3_***	**−3.260**	**0.001**
		**1000 m × *v*_3_ vs. 2300 m × *v_1_***	**13.460**	**<0.001**
		**1000 m × *v*_3_ vs. 2300 m × *v_2_***	**7.050**	**<0.001**
		**1000 m × *v*_3_ vs. 2300 m × *v_3_***	**3.050**	**0.002**
		**2300 m × *v_1_* vs. 2300 m × *v_2_***	**−6.410**	**<0.001**
		**2300 m × *v_1_* vs. 2300 m × *v_3_***	**−10.410**	**<0.001**
		**2300 m × *v_2_* vs. 2300 m × *v_3_***	**−4.000**	**<0.001**
	Speed × Surface	***v*****_1_** **× Road vs. *v*_1_ × Trail**	**−5.04**	**<0.001**
		***v*****_1_** **× Road vs. *v*_2_ × Road**	**−11.03**	**<0.001**
		*v*_1_ × Road vs. *v*_2_ × Trail	1.46	0.147
		***v*****_1_** **× Road vs. *v*_3_ × Road**	**−5.38**	**0.002**
		***v*****_1_** **× Road vs. *v*_3_ × Trail**	**−10.94**	**<0.001**
		***v*****_1_** **× Trail vs. *v*_2_ × Road**	**−5.99**	**<0.001**
		***v*****_1_** **× Trail vs. *v*_2_ × Trail**	**6.5**	**<0.001**
		*v*_1_ × Trail vs. *v*_3_ × Road	−0.34	0.658
		***v*****_1_** **× Trail vs. *v*_3_ × Trail**	**−5.9**	**<0.001**
		***v*****_2_** **× Road vs. *v*_2_ × Trail**	**12.49**	**<0.001**
		***v*****_2_** **× Road vs. *v*_3_ × Road**	**5.65**	**<0.001**
		*v*_2_ × Road vs. *v*_3_ × Trail	0.09	0.910
		***v*****_2_** **× Trail vs. *v*_3_ × Road**	**−6.84**	**<0.001**
		***v*****_2_** **× Trail vs. *v*_3_ × Trail**	**−12.4**	**<0.001**
		***v*****_3_** **× Road vs. *v*_3_ × Trail**	**−5.56**	**<0.001**
Peak tibial acceleration (PTA, *g*)	Elevation × Speed	**50 m × *v*_1_ vs. 50 m × *v*_2_**	**−3.290**	**<0.001**
		**50 m × *v*_1_ vs. 50 m × *v_3_***	**−5.380**	**<0.001**
		50 m × *v*_1_ vs. 1000 m × *v_1_*	0.030	0.882
		**50 m × *v*_1_ vs. 1000 m × *v_2_***	**−2.740**	**<0.001**
		**50 m × *v*_1_ vs. 1000 m × *v_3_***	**−4.640**	**<0.001**
		50 m × *v*_1_ vs. 2300 m × *v_1_*	−0.060	0.865
		**50 m × *v*_1_ vs. 2300 m × *v_2_***	**−2.580**	**<0.001**
		**50 m × *v*_1_ vs. 2300 m × *v_3_***	**−4.170**	**<0.001**
		**50 m × *v*_2_ vs. 50 m × *v_3_***	**−2.090**	**<0.001**
		**50 m × *v*_2_ vs. 1000 m × *v_1_***	**3.320**	**<0.001**
		50 m × *v*_2_ vs. 1000 m × *v_2_*	0.550	0.111
		**50 m × *v*_2_ vs. 1000 m × *v_3_***	**−1.350**	**0.013**
		**50 m × *v*_2_ vs. 2300 m × *v_1_***	**3.230**	**<0.001**
		50 m × *v*_2_ vs. 2300 m × *v_2_*	0.710	0.095
		50 m × *v*_2_ vs. 2300 m × *v_3_*	−0.880	0.088
		**50 m × *v*_3_ vs. 1000 m × *v_1_***	**5.410**	**<0.001**
		**50 m × *v*_3_ vs. 1000 m × *v_2_***	**2.640**	**<0.001**
		50 m × *v*_3_ vs. 1000 m × *v_3_*	0.740	0.125
		**50 m × *v*_3_ vs. 2300 m × *v_1_***	**5.320**	**<0.001**
		**50 m × *v*_3_ vs. 2300 m × *v_2_***	**2.800**	**<0.001**
		**50 m × *v*_3_ vs. 2300 m × *v_3_***	**1.210**	**0.008**
		**1000 m × *v*_1_ vs. 1000 m × *v_2_***	**−2.770**	**<0.001**
		**1000 m × *v*_1_ vs. 1000 m × *v_3_***	**−4.670**	**<0.001**
		1000 m × *v*_1_ vs. 2300 m × *v_1_*	−0.090	0.813
		**1000 m × *v*_1_ vs. 2300 m × *v_2_***	**−2.610**	**<0.001**
		**1000 m × *v*_1_ vs. 2300 m × *v_3_***	**−4.200**	**<0.001**
		**1000 m × *v*_2_ vs. 1000 m × *v_3_***	**−1.900**	**<0.001**
		**1000 m × *v*_2_ vs. 2300 m × *v_1_***	**2.680**	**<0.001**
		1000 m × *v*_2_ vs. 2300 m × *v_2_*	0.160	0.687
		**1000 m × *v*_2_ vs. 2300 m × *v_3_***	**−1.430**	**0.019**
		**1000 m × *v*_3_ vs. 2300 m × *v_1_***	**4.580**	**<0.001**
		**1000 m × *v*_3_ vs. 2300 m × *v_2_***	**2.060**	**0.002**
		1000 m × *v*_3_ vs. 2300 m × *v_3_*	0.470	0.263
		**2300 m × *v_1_* vs. 2300 m × *v_2_***	**−2.520**	**<0.001**
		**2300 m × *v_1_* vs. 2300 m × *v_3_***	**−4.110**	**<0.001**
		**2300 m × *v_2_* vs. 2300 m × *v_3_***	**−1.590**	**<0.001**
	Speed × Surface	***v*****_1_** **× Road vs. *v*_1_ × Trail**	**−3.06**	**<0.001**
		***v*****_1_** **× Road vs. *v*_2_ × Road**	**−5.11**	**<0.001**
		*v*_1_ × Road vs. *v*_2_ × Trail	−0.16	0.355
		***v*****_1_** **× Road vs. *v*_3_ × Road**	**−3.03**	**<0.001**
		***v*****_1_** **× Road vs. *v*_3_ × Trail**	**−5**	**<0.001**
		***v*****_1_** **× Trail vs. *v*_2_ × Road**	**−2.05**	**<0.001**
		***v*****_1_** **× Trail vs. *v*_2_ × Trail**	**2.9**	**<0.001**
		*v*_1_ × Trail vs. *v*_3_ × Road	0.03	0.859
		***v*****_1_** **× Trail vs. *v*_3_ × Trail**	**−1.94**	**<0.001**
		***v*****_2_** **× Road vs. *v*_2_ × Trail**	**4.95**	**<0.001**
		***v*****_2_** **× Road vs. *v*_3_ × Road**	**2.08**	**<0.001**
		*v*_2_ × Road vs. *v*_3_ × Trail	0.11	0.423
		***v*****_2_** **× Trail vs. *v*_3_ × Road**	**−2.87**	**<0.001**
		***v*****_2_** **× Trail vs. *v*_3_ × Trail**	**−4.84**	**<0.001**
		***v*****_3_** **× Road vs. *v*_3_ × Trail**	**−1.97**	**<0.001**
Residual shock (RSh, %)	Elevation × Speed	**50 m × *v*_1_ vs. 50 m × *v*_2_**	**0.1136000**	**<0.001**
		**50 m × *v*_1_ vs. 50 m × *v_3_***	**0.1699800**	**<0.001**
		50 m × *v*_1_ vs. 1000 m × *v_1_*	−0.0130300	0.599
		**50 m × *v*_1_ vs. 1000 m × *v_2_***	**0.0755100**	**0.007**
		**50 m × *v*_1_ vs. 1000 m × *v_3_***	**0.1283200**	**<0.001**
		50 m × *v*_1_ vs. 2300 m × *v_1_*	0.0141900	0.496
		**50 m × *v*_1_ vs. 2300 m × *v_2_***	**0.0927500**	**<0.001**
		**50 m × *v*_1_ vs. 2300 m × *v_3_***	**0.1355700**	**<0.001**
		**50 m × *v*_2_ vs. 50 m × *v_3_***	**0.0563800**	**<0.001**
		**50 m × *v*_2_ vs. 1000 m × *v_1_***	**−0.1266300**	**<0.001**
		50 m × *v*_2_ vs. 1000 m × *v_2_*	−0.0380900	0.066
		50 m × *v*_2_ vs. 1000 m × *v_3_*	0.0147200	0.388
		**50 m × *v*_2_ vs. 2300 m × *v_1_***	**−0.0994100**	**0.005**
		50 m × *v*_2_ vs. 2300 m × *v_2_*	−0.0208500	0.132
		50 m × *v*_2_ vs. 2300 m × *v_3_*	0.0219700	0.188
		**50 m × *v*_3_ vs. 1000 m × *v_1_***	**−0.1830100**	**<0.001**
		**50 m × *v*_3_ vs. 1000 m × *v_2_***	**−0.0944700**	**<0.001**
		**50 m × *v*_3_ vs. 1000 m × *v_3_***	**−0.0416600**	**0.013**
		**50 m × *v*_3_ vs. 2300 m × *v_1_***	**−0.1557900**	**<0.001**
		**50 m × *v*_3_ vs. 2300 m × *v_2_***	**−0.0772300**	**<0.001**
		**50 m × *v*_3_ vs. 2300 m × *v_3_***	**−0.0344100**	**0.018**
		**1000 m × *v*_1_ vs. 1000 m × *v_2_***	**0.0885400**	**<0.001**
		**1000 m × *v*_1_ vs. 1000 m × *v_3_***	**0.1413500**	**<0.001**
		1000 m × *v*_1_ vs. 2300 m × *v_1_*	0.0272200	0.412
		**1000 m × *v*_1_ vs. 2300 m × *v_2_***	**0.1057800**	**0.002**
		**1000 m × *v*_1_ vs. 2300 m × *v_3_***	**0.1486000**	**<0.001**
		**1000 m × *v*_2_ vs. 1000 m × *v_3_***	**0.0528100**	**<0.001**
		1000 m × *v*_2_ vs. 2300 m × *v_1_*	−0.0613200	0.079
		1000 m × *v*_2_ vs. 2300 m × *v_2_*	0.0172400	0.361
		**1000 m × *v*_2_ vs. 2300 m × *v_3_***	**0.0600600**	**0.029**
		**1000 m × *v*_3_ vs. 2300 m × *v_1_***	**−0.1141300**	**0.010**
		1000 m × *v*_3_ vs. 2300 m × *v_2_*	−0.0355700	0.098
		1000 m × *v*_3_ vs. 2300 m × *v_3_*	0.0072500	0.723
		**2300 m × *v_1_* vs. 2300 m × *v_2_***	**0.0785600**	**0.004**
		**2300 m × *v_1_* vs. 2300 m × *v_3_***	**0.1213800**	**0.001**
		**2300 m × *v_2_* vs. 2300 m × *v_3_***	**0.0428200**	**0.008**

### Appendix A.2

#### Appendix A.2.1. Detailed Results on Physiological Control Variables

Regarding HR (Figure A1a), all three main effects, i.e., elevation (*p* < 0.001, *η*_p_^2^ = 0.787), running speed (*p* < 0.001, *η*_p_^2^ = 0.948) and surface (*p* < 0.001, *η*_p_^2^ = 0.776), exhibited strong effects (Table A4). In addition, there are significant interactions between elevation × surface (*p* = 0.045, *η*_p_^2^ = 0.291), elevation × speed (*p* = 0.011, *η*_p_^2^ = 0.299) and elevation × speed × surface (*p* = 0.002, *η*_p_^2^ = 0.361). Only the combination of speed × surface did not show a significant interaction effect on HR (*p* = 0.164; Table A4). Irrespective of the interaction effects, HR, taken as a sole function of altitude, was lower at sea level (160 ± 10 bpm) than at an elevation of 1000 m AMSL (154 ± 10 bpm) or 2300 m AMSL (153 ± 9 bpm; *p* < 0.001), while it was comparable for the latter two (*p* = 0.489; Table A5). Similarly, increasing running speeds resulted in HR rising from 137 ± 12 bpm at *v*_1_ to 158 ± 10 bpm at *v*_2_ and 171 ± 7 bpm at *v*_3_ (*p* < 0.001, Table A5). Furthermore, HR, as averaged across all elevations and speeds, was significantly lower on ROAD (153 ± 9 bpm) compared to TRAIL (158 ± 10 bpm, *p* < 0.001, Table A5). The interaction effects did not provide additional practically relevant insights.

As for LAC (Figure A1b), only running speed (*p* < 0.001, *η*_p_^2^ = 0.862) and surface (*p* = 0.013, *η*_p_^2^ = 0.514) showed significant and strong main effects, whereas elevation did not (*p* = 0.320; Table A4). There were no interaction effects among the three main factors. Post hoc testing showed that LAC was significantly higher at each faster running speed (1.8 ± 0.6, 3.3 ± 1.1 and 6.9 ± 2.1 mmol L^−1^; *p* < 0.001; Table A5). Moreover, LAC was slightly higher on TRAIL when directly compared to ROAD (4.2 ± 1.0 vs. 3.8 ± 1.2 mmol L^−1^; *p* < 0.013, Table A5). Regarding altitude, in contrast, mean LAC, as averaged over all speed and surface conditions, was similar on all elevations (3.8 ± 1.3, 4.0 ± 1.3 and 4.2 ± 0.9 mmol L^−1^ at 50, 1000 and 2300 m AMSL, respectively).

In terms of RPE (Figure A1c), running speed (*p* < 0.001, *η*_p_^2^ = 0.971) was found to exert a strong effect on RPE, whereas elevation (*p* = 0.812) and surface (*p* = 0.051) showed no significant influence (Table A4). There were no pairwise interactions between those main factors (*p* > 0.491), but a significant interaction between elevation × speed × surface (*p* = 0.043, *η*_p_^2^ = 0.234), which did not yield practically relevant insights. Specifically, post hoc comparisons for running speed yielded significantly higher exertion ratings at higher speeds (10.1 ± 1.4 at *v*_1_ vs. 14.6 ± 1.1 at *v*_2_ and 18.3 ± 0.7 at *v*_3_; *p* < 0.001). As a function of elevation, however, RPE was found to remain practically unchanged (14.4 ± 1.1 vs. 14.2 ± 1.2 vs. 14.3 ± 0.8 at 50, 1000 and 2300 m AMSL; *p* = 1.000; Table A5). Similarly, regarding surface, mean RPE averaged across all elevations and running speeds was comparable between ROAD and TRAIL (14.1 ± 0.9 vs. 14.5 ± 1.0) although the trend of a slight difference was approaching the limit of significance (*p* = 0.051; Table A5).

#### Appendix A.2.2. Detailed Discussion of Physiological Control Variables

Our experimental results show that HR was affected strongly by elevation, running speed and surface. However, because HR was used as a control variable for adjusting the running speeds to elicit the same perceived exertion at different altitudes in the employed study design, any interpretation of the relation between HR and elevation can only be of a purely technical nature in terms of model verification. Moreover, it should be noted that the slightly lower temperatures at elevations of 1000 and 2300 m AMSL in our study (approximately 23 °C vs. 17–20 °C) could have possibly biased HR results. This temperature-related effect is typical to real-world altitude scenarios and cannot be easily differentiated from the hypobaric effect on HR, yet plays a noticeable role in exercise practice in altitude training camps. Future research on the complex responses of HR during submaximal efforts in endurance sports under acute altitude-induced hypoxia and associated climatic conditions is thus clearly indicated. Regarding the strong effect of speed on HR, clear increases in HR for faster running were observed, as it was expected from the literature [56]. Despite being slightly smaller, the effect of surface on HR was also strong and comparable to that of elevation in terms of effect size. Furthermore, it was found that HR was higher for a given speed when running on hiking trails compared to paved roads. Although the partly different order of uphill and downhill segments between ROAD and TRAIL conditions (ROAD: more often first downhill; TRAIL: more often first uphill) may have exerted a biassing effect, this observation seems plausible given the comparably narrow, winding and uneven topography of the trails run in this study, which are likely to require more neuromuscular effort for balancing and propulsion when compared to the paved, relatively straight asphalt roads in this study. Nevertheless, by means of the observed average increase of +6 bpm ([+3, +8] bpm; Cohen’s *d* = 0.32), this study may provide a first rough translation formula from road to trail running for practically relevant speeds in amateur runners. Interestingly, however, this increase in HR in trail running still falls in the range of 1–6 bpm that has been reported for the day-to-day intraindividual variance under identical conditions [56].

In terms of RPE, our results show that altitude did not exhibit a statistically significant effect on RPE, confirming that the adjustment of running speeds at altitude conditions was indeed adequate to induce a comparable subjective physiological effort at different elevations, and thus oxygen partial pressures. In view of this, it seems reasonable to postulate that reducing running speeds by roughly −3% per +1000 m in elevation gain accounts for a—subjectively—higher physiological effort at altitude. For a running pace of, e.g., 4:15 min km^−1^ this would imply a reduction in pace to 4:30 min km^−1^ at 2000 m AMSL, thus approximately 15 s, which is in good quantitative agreement with current coaching procedures. In terms of the effect of surface, the study outcome suggests that the athletes did not perceive trail or road conditions as a particularly strenuous factor for the submaximal running speeds given.

As for LAC, higher running speeds resulted in higher levels, as suggested by the underlying metabolic processes below, at and between the ventilatory thresholds 1 and 2 and widely expected from the literature [57,58]. Trail running induced slightly lower LAC levels than road running, with an average drop of −0.4 ([−0.7, −0.1]) mmol L^−1^ and an associated negligible effect size of Cohen’s *d* = −0.15. As above, it should be noted in this context that the different order of uphill and downhill segments between ROAD and TRAIL conditions may have contributed to this result as a confounding factor because lactate accumulated during initial uphill running may have been partially cleared during the consecutive downhill segment in TRAIL. Moreover, elevation did not significantly affect LAC accumulation, yielding similar LAC levels at altitude compared to sea level. This outcome is in accordance with the results reported in the literature [8,15], particularly for the acute, short-term exposure to hypoxia administered to the subjects of this study, where no effect of the “lactate paradox” could be expected. It is noted that the tendency to slightly lower LAC levels at altitude in this study is consistent with the trend reported by Friedmann et al. in their laboratory-based trial [15], although the lack of statistical significance in either set of results recommends against further interpretation.

**Table A4:** Physiological control variables at end of runs along with the corresponding main effects of a three-way rmANOVA.

Load Metric	Elevation AMSL	Speed	Surface		Significance Level *p* and Effect Size *η*_p_^2^						
			ROAD	TRAIL	Elevation	Speed	Surface	Elevation × Surface	Elevation × Speed	Speed × Surface	Elevation × Speed × Surface
Heart rate (HR, bpm)	50 m	*v*_1_	140 ± 14	141 ± 15	***p*** **< 0.001**	***p*** **< 0.001**	***p*** **< 0.001**	***p*** **= 0.045**	***p*** **= 0.011**	*p* = 0.164	***p*** **= 0.002**
		*v*_2_	161 ± 10	165 ± 11							
		*v*_3_	174 ± 8	178 ± 8							
	1000 m	*v*_1_	131 ± 13	139 ± 13							
		*v*_2_	153 ± 11	160 ± 10							
		*v*_3_	168 ± 8	173 ± 7							
	2300 m	*v*_1_	131 ± 12	141 ± 11	***η*****_p_****^2^** **= 0.787**	***η*****_p_****^2^** **= 0.948**	***η*****_p_****^2^** **= 0.776**	***η*****_p_****^2^** **= 0.291**	***η*****_p_****^2^** **= 0.299**	*η*_p_^2^ = 0.199	***η*****_p_****^2^** **= 0.361**
		*v*_2_	152 ± 9	160 ± 10							
		*v*_3_	166 ± 8	169 ± 7							
Blood lactate concentration (LAC, mmol L^−1^)	50 m	*v*_1_	1.6 ± 1.5	1.5 ± 0.7	*p* = 0.320	***p*** **< 0.001**	***p*** **= 0.013**	*p* = 0.337	*p* = 0.980	*p* = 0.165	*p* = 0.661
		*v*_2_	2.4 ± 1.1	3.6 ± 1.7							
		*v*_3_	6.1 ± 2.4	7.4 ± 2.7							
	1000 m	*v*_1_	2.0 ± 1.3	1.8 ± 0.9							
		*v*_2_	3.2 ± 1.6	3.6 ± 1.4							
		*v*_3_	6.5 ± 3.1	7.2 ± 2.3							
	2300 m	*v*_1_	2.0 ± 0.6	1.9 ± 0.4	*η*_p_^2^ = 0.119	***η*****_p_****^2^** **= 0.862**	***η*****_p_****^2^** **= 0.514**	*η*_p_^2^ = 0.114	*η*_p_^2^ = 0.011	*η*_p_^2^ = 0.181	*η*_p_^2^ = 0.039
		*v*_2_	3.4 ± 0.9	3.7 ± 1.0							
		*v*_3_	6.9 ± 2.4	7.2 ± 2.8							
Rating of perceived exertion (RPE; Borg 6–20)	50 m	*v*_1_	9.8 ± 1.7	10.4 ± 2.0	*p* = 0.812	***p*** **< 0.001**	*p* = 0.051	*p* = 0.525	*p* = 0.491	*p* = 0.695	***p*** **= 0.043**
		*v*_2_	14.4 ± 1.4	15.4 ± 1.8							
		*v*_3_	18.0 ± 0.8	18.5 ± 0.8							
	1000 m	*v*_1_	10.2 ± 2.2	10.0 ± 1.9							
		*v*_2_	14.4 ± 1.1	14.3 ± 1.6							
		*v*_3_	18.0 ± 0.9	18.5 ± 1.2							
	2300 m	*v*_1_	9.6 ± 1.5	10.4 ± 2.0	*η*_p_^2^ = 0.023	***η*****_p_****^2^** **= 0.971**	*η*_p_^2^ = 0.359	*η*_p_^2^ = 0.069	*η*_p_^2^ = 0.077	*η*_p_^2^ = 0.040	***η*****_p_****^2^** **= 0.234**
		*v*_2_	14.6 ± 1.0	14.2 ± 2.0							
		*v*_3_	18.3 ± 0.9	18.4 ± 1.0							

Comments: The cohort size was *n* = 10 participants. All outcome values are presented as means ± standard deviations. Significance levels *p* and effect sizes η_p_^2^ are based on a three-way repeated-measures analysis of variance (rmANOVA; see text). Significant results are highlighted in boldface.

**Table A5:** Post hoc comparisons for the physiological control variables at the end of runs (three-way rmANOVA).

Load Metric	Factor	Post Hoc Testing	Mean Difference (95% CI)	Significance Level
Heart rate (HR, bpm)	Elevation	**50 vs. 1000 m AMSL**	**5.6 (2.4 to 8.8)**	***p*** **= 0.002**
		**50 vs. 2300 m AMSL**	**6.8 (4.5 to 9.0)**	***p*** **< 0.001**
		1000 vs. 2300 m AMSL	1.2 (−1.1 to 3.8)	*p* = 0.489
	Speed	***v*****_1_** **vs. *v*_2_**	**−21 (−25 to −17)**	***p*** **< 0.001**
		***v*****_1_** **vs. *v*_3_**	**−34 (−42 to −26)**	***p*** **< 0.001**
		***v*****_2_** **vs. *v*_3_**	**−12 (−17 to −9)**	***p*** **< 0.001**
	Surface	**Road vs. Trail**	**−5.7 (3.4 to 7.9**	***p*** **< 0.001**
Blood lactate concentration (LAC, mmol L^−1^)	Elevation	50 vs. 1000 m AMSL	−0.3 (−1.0 to 0.4)	*p* = 0.814
		50 vs. 2300 m AMSL	−0.4 (−1.4 to 0.5)	*p* = 0.698
		1000 vs. 2300 m AMSL	−0.1 (−0.9 to 0.6)	*p* = 1.000
	Speed	***v*****_1_** **vs. *v*_2_**	**−1.5 (−2.4 to −0.7)**	***p*** **= 0.002**
		***v*****_1_** **vs. *v*_3_**	**−5.1 (−7.0 to −3.2)**	***p*** **< 0.001**
		***v*****_2_** **vs. *v*_3_**	**−3.6 (−4.9 to −2.2)**	***p*** **< 0.001**
	Surface	**Road vs. Trail**	**−0.4 (−0.7 to −0.1)**	***p*** **= 0.013**
Rating of perceived exertion (RPE, Borg 6–20)	Elevation	50 vs. 1000 m AMSL	0.2 (−0.5 to 0.9)	*p* = 1.000
		50 vs. 2300 m AMSL	0.2 (−0.9 to 1.2)	*p* = 1.000
		1000 vs. 2300 m AMSL	−0.02 (−1.0 to 1.0)	*p* = 1.000
	Speed	***v*****_1_** **vs. *v*_2_**	**−4.5 (−5.1 to −3.9)**	***p*** **< 0.001**
		***v*****_1_** **vs. *v*_3_**	**−8.2 (−9.5 to −7.0)**	***p*** **< 0.001**
		***v*****_2_** **vs. *v*_3_**	**−3.7 (−4.7 to −2.7)**	***p*** **< 0.001**
	Surface	Road vs. Trail	−0.3 (−0.6 to 0.002)	*p* = 0.051

Comments: The cohort size was *n* = 10 participants. Significance levels *p* and confidence intervals (CI) were derived from Bonferroni-corrected post-hoc testing for the three-way repeated-measures analysis of variance (rmANOVA; see text). Significant results are highlighted in boldface.
